# Experiences of pelvic floor dysfunction and treatment in women with breast cancer: a qualitative study

**DOI:** 10.1007/s00520-022-07273-2

**Published:** 2022-07-05

**Authors:** Udari N. Colombage, Kuan-Yin Lin, Sze-Ee Soh, Robyn Brennen, Helena C. Frawley

**Affiliations:** 1 Department of Physiotherapy, Melbourne School of Health Sciences, Faculty of Medicine, Dentistry & Health Sciences, The University of Melbourne, Melbourne, Australia; 2 School of Health, Federation University, Churchill, Australia; 3 Department of Physical Therapy, National Cheng Kung University, Tainan, Taiwan; 4 Institute of Allied Health Sciences, College of Medicine, National Cheng Kung University, Tainan, Taiwan; 5 Department of Physiotherapy, Monash University, Melbourne, Australia; 6 Department of Epidemiology and Preventive Medicine, Monash University, Melbourne, Australia; 7 Specialist Clinics, Monash Health, Cheltenham, Australia; 8 Allied Health Research, Royal Women’s Hospital, Melbourne, Australia; 9 Allied Health Research, Mercy Hospital for Women, Melbourne, Australia

**Keywords:** Breast cancer, Pelvic floor dysfunction, Cancer treatement

## Abstract

**Purpose:**

To explore the experiences of women with breast cancer and pelvic floor (PF) dysfunction and the perceived enablers and barriers to uptake of treatment for PF dysfunction during their recovery.

**Method:**

Purposive sampling was used to recruit 30 women with a past diagnosis of breast cancer and PF dysfunction. Semi-structured interviews were conducted, and data were analysed inductively to identify new concepts in the experiences of PF dysfunction in women with breast cancer and deductively according to the capability, opportunity, motivation and behaviour (COM-B) framework to identify the enablers and barriers to the uptake of treatment for PF dysfunction in women with breast cancer.

**Results:**

Participants were aged between 31 and 88 years, diagnosed with stages I–IV breast cancer and experienced either urinary incontinence (*n* = 24/30, 80%), faecal incontinence (*n* = 6/30, 20%) or sexual dysfunction (*n* = 20/30, 67%). They were either resigned to or bothered by their PF dysfunction; bother was exacerbated by embarrassment from experiencing PF symptoms in public. Barriers to accessing treatment for PF dysfunction included a lack of awareness about PF dysfunction following breast cancer treatments and health care professionals not focussing on the management of PF symptoms during cancer treatment. An enabler was their motivation to resume their normal pre-cancer lives.

**Conclusion:**

Participants in this study reported that there needs to be more awareness about PF dysfunction in women undergoing treatment for breast cancer. They would like to receive information about PF dysfunction prior to starting cancer treatment, be screened for PF dysfunction during cancer treatment and be offered therapies for their PF dysfunction after primary cancer treatment. Therefore, a greater focus on managing PF symptoms by clinicians may be warranted in women with breast cancer.

## Introduction

Breast cancer is the most common cancer affecting women in Australia [1]. Despite the growing incidence of breast cancer, mortality rates are decreasing due to advancements in breast cancer treatments [2]. Recent literature reported that women with breast cancer may experience pelvic floor (PF) dysfunction including urinary incontinence (UI), faecal incontinence (FI) and sexual dysfunction after receiving treatment for breast cancer [3, 4]. The development of these symptoms in women with breast cancer may be related to oestrogen depletion due to ovarian suppression and failure after chemotherapy or endocrine therapy use [5]. Pelvic floor symptoms appear to be more severe in women with breast cancer than postmenopausal women without breast cancer due to the prolonged and higher degree of hypoestrogenism caused by breast cancer treatments compared to natural menopause [6]. One study highlighted that at least 25% of women experienced new or worsened UI within three months of receiving neoadjuvant chemotherapy [7]. Coupled with this, women with breast cancer may adopt a more sedentary lifestyle during recovery which may be associated with the development and worsening of PF symptoms [8]. While current breast cancer treatment guidelines highlight the importance of providing support for sexual dysfunction after breast cancer, the screening and management of UI and FI do not appear in breast cancer pathways of care [9, 10] and often go untreated in this population [11]. Therefore, it is important to understand the symptom burden of PF dysfunction in women with breast cancer in order to inform care.

Data from quantitative studies have shown that women with breast cancer may experience a higher impact of PF dysfunction than women without breast cancer [3, 12]. Recent data also indicate that the impact of PF dysfunction increases as women move on from their breast cancer diagnosis and enter the survivorship phase of their recovery (i.e. five years post-cancer diagnosis) [13]. This may be due to unmet survivorship needs such as sexual and bladder dysfunction that are often under-addressed by healthcare professionals [14]. However, there are limited qualitative studies conducted on the experiences of PF dysfunction in women with breast cancer [15, 16]. One study exploring the experiences of genitourinary symptoms in women with breast cancer reported that these symptoms were often a source of embarrassment and negatively impacted on the lives of women [15]. Another qualitative study in this population highlighted that some women had an emotional crisis when experiencing such symptoms after breast cancer treatment [16]. These studies however highlighted that the impacts of PF symptoms in women on long-term breast cancer treatments are unclear [15, 16]. The impacts of PF dysfunction may differ at various points of the breast cancer treatment trajectory (e.g. newly diagnosed, after active cancer treatment or after meeting the five-year survivorship milestone). A study on women without breast cancer reported that women often modify the activities of daily living and reduce social interactions to conceal and control symptoms of PF dysfunction [17]. Symptoms of PF dysfunction may therefore be a major barrier to resuming life after cancer recovery in women with breast cancer. Therefore, these time-point distinctions may illuminate the experiences of PF dysfunction women may have in the short, middle and longer-term after a breast cancer diagnosis. The impact of these symptoms is important to know in order to know at what time point in their breast cancer trajectory PF symptoms become burdensome. Qualitative studies also provide insights into women’s enablers and barriers to seeking and taking up treatment for their PF dysfunction.

The capability, opportunity, motivation and behaviour (COM-B) framework [18] is widely used to understand human behaviour associated with the enablers and barriers to engaging with treatment [19]. This framework states that human behaviour is impacted by psychological and physical capabilities, social opportunities and individual motivators [18]. These factors are important to address when designing treatments (such as PF muscle training for PF dysfunction), testing the efficacy of treatments and implementing new therapies into clinical care pathways [20]. Thus, this study aimed to explore the experiences of Australian women with breast cancer who have PF dysfunction and the perceived enablers and barriers to the uptake of treatment for PF dysfunction during their breast cancer treatment trajectory, mapped against the COM-B framework. In this study, we considered participant experiences to be personal interpretations and recall of phenomena in their lives [21]. We also explored when women want to receive information and treatment for PF dysfunction.

## Methods

This study is reported according to the COnsolidated criteria for REporting Qualitative studies (COREQ) [22]. This research was approved by the University of Melbourne Office of Research Ethics and Integrity (ID: 22,462).

### Personal characteristics

The first author completed interviews via videoconference (Zoom™). The interviewer was a female, registered physiotherapist and a PhD candidate with experience in conducting research on PF dysfunction in women with breast cancer. She had received training in qualitative research to inform the design of this project.

### Relationship with participants

None of the researchers had an established relationship with any participants prior to the commencement of the study. Details about the research project including the research aims and experience of the research team were outlined in the explanatory statement.

### Theoretical framework

This study was underpinned by thematic analysis based on a descriptive phenomenological approach [23]. This framework seeks to explore how people experience a specific phenomenon and allows researchers to find meaning according to the trends and patterns found in the experiences of people [24]. It is a popular approach that can be used to explore the lived experience of patients with cancer by encouraging researchers to set aside preconceived assumptions about the lives of participants. [24]

### Participant selection

A study flyer was posted online by various social media groups that connected with women with breast cancer. Women expressed their interest to participate in the study by completing an eligibility screening questionnaire. Eligible participants were contacted by a researcher to schedule a time for their interview and to complete an online questionnaire containing the explanatory statement, consent form and demographic questions. Purposive sampling was used to recruit a total of 30 women into three groups (each group *n* = 10). Eligibility criteria for each group are listed below.

- Group 1 – Currently undergoing primary cancer treatment: women over the age of 18 diagnosed with any stage of breast cancer who were undergoing primary cancer treatment and currently experienced PF dysfunction.
- Group 2 – Completed primary cancer treatment: women over the age of 18 diagnosed with any stage of breast cancer who had completed primary cancer treatment and currently experienced PF dysfunction. They may still be undergoing adjuvant cancer treatment.
- Group 3 – Reached survivorship milestone: women over the age of 18 diagnosed with any stage of breast cancer who had reached their survivorship milestone (i.e. diagnosed five or more years ago) and currently experienced PF dysfunction. They may still be undergoing adjuvant cancer treatment.

### Setting

One-on-one interviews were conducted via videoconference (Zoom™). Only the participant and researcher were present during the interview. Videoconferencing has been a popular method for qualitative data collection in the pandemic era as it has a number of advantages such as being able to maintain social distance, removing the need for travel and being able to connect with participants in geographically distant location. [25]

### Data collection

Demographic and medical data including age, stage of breast cancer, year of breast cancer diagnosis, height, weight, parity, menopausal status at the time of data collection, any breast cancer treatments received and current PF symptoms were collected using online questionnaires. The presence of PF dysfunction was established using the following screening questions: “UI is the accidental loss of bladder control. Do you currently experience UI?”; “FI is the accidental loss of bowel control. Do you currently experience FI?”; and “Sexual dysfunction is difficulty experienced during any stage of sexual activity. This may include painful intercourse or problems with sexual desire, arousal or orgasm that cause distress. Do you currently experience sexual dysfunction?”.

A semi-structured interview guide was developed (see Appendix) and piloted with consumer collaborators prior to the commencement of the study. Changes to the wording of questions in the semi-structured interview guide were made in response to piloting (e.g. explanation of PF physiotherapy). During the interview, participants were asked about their experiences of PF dysfunction and their perceived enablers and barriers to treatment for PF dysfunction. All interviews were audio-recorded and transcribed. The transcripts were returned to participants to make additional comments for checking the accuracy of the interview.

### Data analysis

Socio-demographic and medical data were presented descriptively. Interview data were transcribed verbatim by an external transcriber and uploaded to NVivo 1.3 (QSR International, Doncaster, Australia). Qualitative data from interviews were analysed using thematic analysis. Inductive coding was applied to identify new concepts in the experiences of PF dysfunction in women with breast cancer, and deductive coding was applied using the three COM-B domains to identify the enablers and barriers to the uptake of treatment for PF dysfunction in women with breast cancer. Emerging themes were initially independently coded, and the codes were checked by another author. Any disagreements were discussed with the research team until a consensus was reached. Data collection continued until data saturation was achieved.

## Results

### Participant characteristics

All interviews were conducted between January and July 2021 and ranged from 20 to 65 min in duration (mean of 37 min). All participants were at home during the interview. Participants were aged between 31 and 88 years, diagnosed with stages I–IV breast cancer and experienced urinary incontinence (24/30, 80%), faecal incontinence (6/30, 20%) or sexual dysfunction (20/30, 67%). Table 1 presents the participant characteristics according to the three groups of women interviewed.

**Table 1:** Participant characteristics and medical history according to interviewed groups

Characteristics	All women (*n* = 30)	Group 1 (*n* = 10)	Group 2 (*n* = 10)	Group 3 (*n* = 10)
Age, years	50 (9)	52 (14)	50 (7)	50 (8)
Australian state or territory, *n* (%)				
Australian Capital Territory	2 (7)	1 (10)	1 (10)	0 (0)
New South Wales	6 (20)	2 (20)	2 (20)	2 (20)
Northern Territory	1 (3)	0 (0)	1 (10)	0 (0)
Queensland	4 (13)	1 (10)	1 (10)	2 (20)
South Australia	4 (13)	3 (30)	1 (10)	0 (0)
Tasmania	2 (7)	1 (10)	0 (0)	1 (10)
Victoria	6 (20)	1 (10)	2 (20)	3 (30)
Western Australia	5 (17)	1 (10)	2 (20)	2 (20)
Body mass index, kg/m^2^	30 (5)	29 (6)	30 (4)	30 (5)
Parity	2 (1)	1.5 (1)	2 (1)	2.5 (1)
Years since diagnosis	4 (3)	1 (1)	3 (1)	8 (3)
Menopausal status, *n* (%)				
Pre	1 (3)	1 (10)	0 (0)	0 (0)
Peri	8 (27)	2 (20)	3 (30)	3 (30)
Post	21 (70)	7 (70)	7 (70)	7 (70)
Breast cancer stage, *n* (%)				
I	2 (6)	1 (10)	1 (10)	0 (0)
II	12 (40)	6 (60)	4 (40)	2 (20)
III	11 (37)	3 (30)	2 (20)	6 (60)
IV	5 (17)	0 (0)	3 (30)	2 (20)
Breast cancer treatment, *n* (%)*				
Surgery	24 (80)	5 (50)	9 (90)	10 (100)
Chemotherapy	23 (77)	8 (80)	7 (70)	8 (80)
Radiotherapy	20 (67)	5 (50)	6 (60)	9 (90)
Tamoxifen	13 (43)	3 (30)	4 (40)	6 (60)
Aromatase inhibitors	14 (47)	5 (50)	4 (40)	5 (50)
Presence of pelvic floor dysfunction, *n* (%)*				
Urinary incontinence	24 (80)	7 (70)	8 (80)	9 (90)
Faecal incontinence	6 (20)	2 (20)	3 (30)	1 (10)
Sexual dysfunction	20 (67)	7 (70)	6 (60)	7 (70)

Group 1, currently undergoing primary cancer treatment. Group 2, completed primary cancer treatment. Group 3, reached survivorship milestone

All data presented are mean (standard deviation) unless stated otherwise

^*^Multiple options were allowed to be selected

### Experiences of pelvic floor dysfunction

All of our participants were either resigned to or bothered by their PF dysfunction. Those who were resigned felt their PF dysfunction was a low priority compared to other difficulties they faced during their cancer treatment.

- “A low priority. I don’t really feel that it’s, I mean, yes, it is an issue, but by the scheme of things. I’ve got bigger issues with the cancer. I just kind of deal with that [incontinence] as a side effect.” – Age 52.

Bother was driven by the embarrassment of experiencing PF symptoms when in public.

- “Whenever I’m out and about if I’m too far from home. I worry that oh am I going to be that person that’s going to have to like squat on the side of the road or find a park or a tree or something? And it’s horrible, it’s embarrassing and I try to hide the mess.” – Age 51.
- “You can hide if you have urinary incontinence but the bowel one is more obvious. Especially if it happens in public, yeah, you might stink in front of people. That’s not good… I mean, there’s nothing worse if you’re running around and you just go.” – Age 43.

Participants’ reactions to the experiences of PF dysfunction (i.e. feeling resigned to or bothered) contributed to acting as a barrier or enabler to the uptake of treatment for PF dysfunction and are further explained in the next section.

### Experiences related to the uptake of treatment for pelvic floor dysfunction

Barriers to the uptake of treatment for PF dysfunction have been mapped according to the COM-B model. Participants reported that their lack of awareness that PF dysfunction may follow breast cancer treatment, the decision not to disclose PF symptoms to health care professionals, belief that health care professionals do not focus on PF symptoms, belief that PF physiotherapy is difficult to access and low prioritisation of PF symptoms were the major barriers to the uptake of treatment for PF dysfunction. The five themes related to barriers did not differ across the three groups of participants.

### Capability barriers

#### Lack of awareness that pelvic floor dysfunction may follow breast cancer treatment

Many participants reported that they were unaware of PF dysfunction following breast cancer treatment. They felt that the possibility of this should have been better communicated to them by health care professionals so that PF symptoms could be addressed early.

- “And no one actually sits down and says, okay, you’re going to have chemo, you’re gonna have radiation, and this is what’s going to happen. This [urinary incontinence] is a possibility. Now, if this happens, you need to go and speak to somebody about it. This is that but that’s never brought up. Nothing’s ever brought up, well it wasn’t with me. About what happens post treatment.” – Age 37.

### Opportunity barriers

#### Not disclosing pelvic floor symptoms to health care professionals

Some participants felt that PF dysfunction was an embarrassing topic to discuss with health care professionals. This opinion was shared by younger women (aged 20–40 years) as they felt it was uncommon to experience these symptoms at their age.

- “Most breast cancer survivors don’t talk about it, because well, why would you? It’s kind of embarrassing and shameful, that you’ve lost your sex life, and like, you’re incontinent in your 40 s.” – Age 43.

#### Belief that health care professionals do not focus on pelvic floor symptoms

Some participants felt that their concerns about PF symptoms were ignored by health care professionals. Despite expressing their concerns about these symptoms to their cancer care team, participants reported that health care professionals tended to preferentially focus on cancer treatment and recovery, rather than address their concerns about PF dysfunction.

- “I just feel like they don’t really care about these things. They focus on like killing the cancer and then that’s it, good bye. They haven’t offered anything else for it.” – Age 54.
- “The male oncologists that I had just really didn’t take any of it seriously at all. Like they didn’t want to, and I’m busy having arguments with them saying, you know what, I actually can’t live like this…we’ve actually got to do something here. There’s been a few times where I would actually rather be dead than keep living like this.” – Age 51.
- “Well, basically they said, because I was going through all the stuff with the cancer. It’s like any other medical stuff when you have cancer. It’s get this cancer stuff sorted out first. And then once everything’s sorted out, then we’ll deal with everything else as it comes up.” – Age 59.

Furthermore, participants in some states of Australia felt that there was a lack of follow-up care and support services after their primary cancer treatment. Many participants (*n* = 23) reported that they were not provided with information nor support regarding how to manage their PF dysfunction.

- “This [breast cancer] has happened. Surgery happens, radiation happens, chemo that happens. And then it stop[s]. Silence. Nothing.” – Age 44.
- “Like if you’re going to do that much crap to a person, you should really be providing that much support afterwards.” – Age 47.
- “I spoke to my doctor about the sex thing. And she was like, how most doctors are, Oh yes, go to the pharmacy, and you’ll be able to get something. She wasn’t forthcoming, on offering me anything more than what I could get at the pharmacy. And when that happens, you don’t want to harp on about it.” – Age 52.

#### Belief that pelvic floor physiotherapy is difficult to access

Some participants reported the high cost, limited availability of PF physiotherapy appointments and having limited time as barriers to accessing physiotherapy to treat PF dysfunction.

- “People might avoid doing that [physiotherapy], because it costs money. And I mean, breast cancer costs a lot of money.” – Age 49.
- “There is what appears to be limited, women’s health physiotherapists and then access and actually getting an appointment. In my area, when I was trying to look up women’s health physios, there was one.” – Age 31.
- “I think just being time poor, you know, with the amount of appointments that we do have. So, you know, just having to fit it in, it’s just it’s another thing that you have to fit in.” – Age 52.

### Motivation barriers

#### Low prioritisation of PF symptoms

Some participants commented that they felt resigned to their PF symptoms compared to their recent breast cancer experience.

- “I think because I have metastatic breast cancer. And I think that in the scheme of things, like having something that makes me uncomfortable and embarrassed, it’s just not gonna let it ruin my day.” – Age 58.

Others reported that their primary concern was to focus on cancer treatment and recovery. Therefore, getting treatment for their PF symptoms was not a priority. This was primarily reported in participants currently undergoing treatment for breast cancer (group 1). They felt that their PF dysfunction would resolve on its own after the completion of active cancer treatment.

- “You’ve got cancer, you’ve got chemo, and then you have 20 leaflets in front of you. And you’re thinking, oh my god. Focus on getting through the cancer part… So that part of you is like that’s [incontinence is] something I’ll deal with that later, I’m gonna get used to having one breast or no nipples, or whatever it is that you have to deal with first.” – Age 39.
  “It’s like a side effect of chemo, and hopefully like the rest of the side effects it, it goes away over time… So I’m kind of hoping that now that I almost finished treatment, it's going to go back to normal.” – Age 42.

Enablers to the uptake of treatment for PF dysfunction have also been mapped according to the COM-B model. Participants reported that drawing on previous experience with PF physiotherapy to self-manage PF symptoms, already having accessed physiotherapy as a part of breast cancer treatment, being interested in accessing physiotherapy via telehealth and their motivation to get back to their pre-cancer lives were the main enablers to the uptake of treatment for PF dysfunction. These four themes did not differ between the three groups.

### Capability enablers

#### Draw on previous experience with pelvic floor physiotherapy to self-manage pelvic floor symptoms

Participants who had accessed PF physiotherapy services prior to breast cancer treatment continued to do PF muscle exercises themselves. However, they felt it would have been beneficial to be guided by health professionals on these PF muscle exercises to confirm their technique and effectiveness.

- “I know some basic pelvic floor exercises, and I did and still do those. But I know there’s an awful lot more that a professional can do and will know how to target effectively.” – Age 48.

### Opportunity enablers

#### Already accessing physiotherapy as a part of breast cancer treatment

Participants who had finished primary treatment and were focussing on cancer recovery (group 2) felt that PF physiotherapy should be integrated into existing breast cancer physiotherapy treatments.

- “They could incorporate it into that and say okay ladies we are going to do exercises that’s going to help you regain your flexibility in your shoulders and your arms and your chest, but also, we are going to have a talk about pelvic floor exercises because some of you may experience it. So that would also be a good time, that would be a great opportunity.” – Age 43.
- “If they’re gonna take you in there and talk to you about lymphedema, it’s the perfect opportunity for them to be discussing this other side effects [incontinence] as well.” – Age 56.

#### Interested in accessing physiotherapy via telehealth

Some participants commented that the option of accessing PF physiotherapy via telehealth would provide them with a number of benefits such as better accessibility, time efficiency and privacy.

- “If it’s in their, if it’s in the comfort of your own home via Zoom, then obviously, that makes it a lot more accessible to women. And convenient too.” – Age 59.
- “I’ve been able to do a lot of appointments via telehealth which is saving me a 40 min drive. And, you know, then sitting around, it’s just like, here I am. Yeah, it’s so much easier.” – Age 38.
- “If I could use telehealth yeah… Because talking about those bottom bits you want it to be more private at home.” – Age 47.

### Motivation enablers

#### Get back to pre-cancer lifestyle

The motivation for many participants in accessing treatment for PF dysfunction was to resume their pre-cancer lives and activities. Some participants also feared that their PF dysfunction would get worse without treatment.

- “I want to be able to get out and not worry about anything [incontinence] anymore. I want to drive up and go out into the open or go down the beach.” – Age 45.
- “I’ve spent three years surviving, and I don’t want to survive for the next 25 years…So much of you gets ripped away, and the old you disappears. This [incontinence] is such a big part of that. You know who you were, and having that back, it will go a heck of a long way to feeling like life is more normal. And get a bit more of you back… Not survivorship but thrive-rship.” – Age 50.
- “I don’t want thing getting worse. I’m very, very particular about it. That would, that’s probably what would bother me more than anything, you know, I don’t want to walk around with things like that, I think I’d rather shoot myself than have those sorts of issues. It’s not nice.” – Age 52.

### Timing of information and treatment offered for pelvic floor dysfunction

**Fig. 1:**
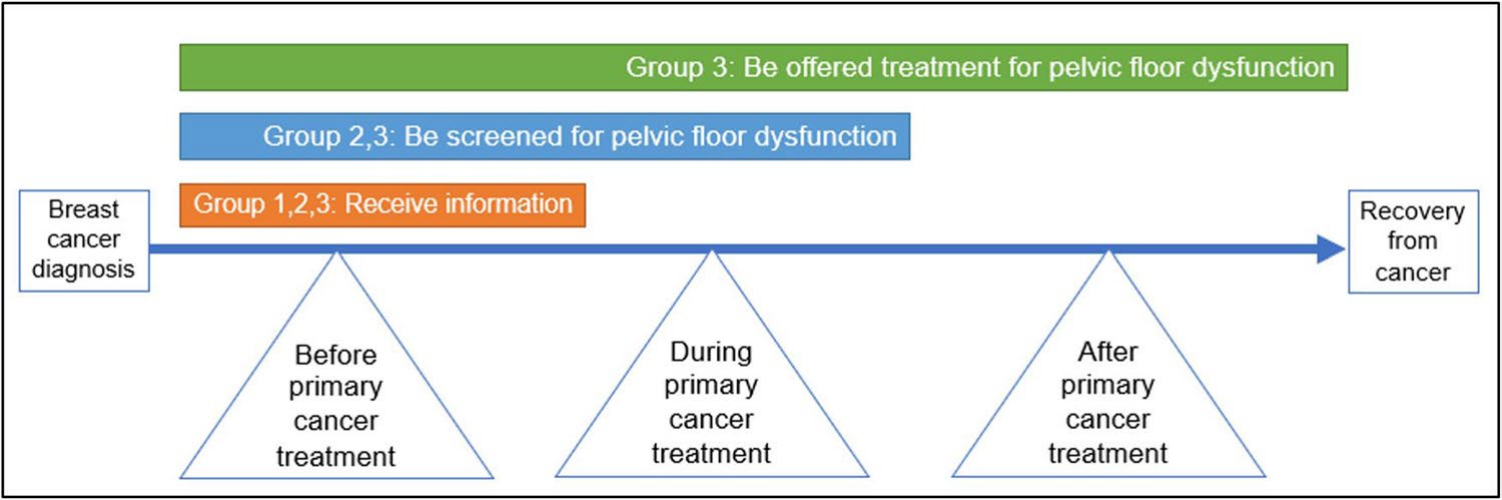
Participant preferences on the best timing to address pelvic floor dysfunction across the breast cancer treatment trajectory

In all groups, only participants who expressed bother about their PF dysfunction were interested to seek treatment for PF dysfunction. These participants wanted to receive information about PF dysfunction as a result of breast cancer treatment before commencing primary cancer treatment. However, there were differences in opinion between groups about the best time to be screened and offered treatment for PF dysfunction through the breast cancer treatment trajectory (Fig. 1).

Participants who were currently undergoing primary cancer treatment (group 1) felt that while it is important to get that information at the start of their cancer treatment trajectory, they would seek treatment for PF dysfunction towards the end of their cancer treatment trajectory.

- “I think before [primary cancer treatment] there’s so much going on, there’s so many treatments going on. That’s [incontinence is] probably not on your mind at the moment at that time. But most certainly after [primary cancer treatment] when you’ve come back down to earth and it’s time to get on with normal life. And you know, you’re not getting on with normal life, because things that you were doing before aren’t happening now. So I think definitely, it needs to be addressed after.” – Age 53.
- “It [incontinence] should be something that’s mentioned right up front. I think that we’re not given all of the information about the side effects… Maybe there needs to be something in the breast cancer books that talks about these issues that you’re probably going to face. They have lots of pamphlets from the Cancer Council, you know, information about all different side effects. So maybe there could be something about urinary incontinence in there”. – Age 43.

The majority of participants who had just finished primary cancer treatment (group 2) wished for more information about PF dysfunction at an earlier stage. They suggested that more screening and open discussions about PF symptoms by health care professionals during primary cancer treatment would be beneficial.

- “Screen for it, ask her if she has a problem with it…It will be good if there is some specific little thing within the whole breast cancer treatment where it’s like, well if you are having issues with bowels, urinary, or sexual, come speak to us. I wish they offered that to us during cancer treatment so that we can deal with it then. Together with the cancer.” – Age 52.
- “In the early stages, when you’re in that primary treatment. You get a lot of information thrown at you and some of it you go, okay, that’s for later, but you still keep that little nugget that I got something about that, and then you go and pull it out when you’re ready for it. So I think it is having that… Arming people with tools and knowledge on what options are open to them and what they can do and how to tackle it.” – Age 39.

Most participants who had reached their survivorship milestone (group 3) reported that they wished there was more awareness and screening of PF dysfunction during their primary treatment for them to access treatment for PF dysfunction earlier. They felt that it is important to address PF symptoms early, through all stages of cancer treatment.

- “No one told me that incontinence could happen as a result of cancer treatment. So if I was aware of it, that it was a side effect, then I would have started to see someone about it when it happened. Yeah. I would have done something about it earlier…even during or after [cancer] treatment. Because now I feel it’s too late.” – Age 43.

## Discussion

This study found that our participants with PF dysfunction were either bothered by (driven by embarrassment) or resigned to (assigned a low priority) PF symptoms during their breast cancer treatment and recovery. These reactions to the experiences of PF symptoms contributed to the barriers and enablers impacting on the uptake of treatment for PF dysfunction. Overall, participants expressed that they would like to receive information about PF dysfunction prior to starting cancer treatment, be screened for PF dysfunction during cancer treatment and be offered therapies for their PF dysfunction right after primary cancer treatment. These results indicate that a greater focus on managing PF symptoms by clinicians may be warranted in women with breast cancer.

Our participants who were bothered by their PF symptoms expressed that their bother was driven by the embarrassment of experiencing PF symptoms in public settings. Similar concerns have been reported by studies conducted in women with gynaecological cancer [26] and women without cancer [27] who experienced embarrassment, stress and low quality of life due to UI. These studies highlighted that participants worried about their PF symptoms being noticed by others due to marks on clothing or unpleasant odours when in public, which ultimately decreased their interest to attend social events outside their homes or in familiar settings [26, 27]. Active social engagement is encouraged in women with breast cancer due to its associated benefits for their physical and mental health and reduced breast cancer-related mortality [28]. Therefore, the treatment of PF symptoms has the potential to encourage social engagement and allows women to more actively participate in society and improve their health and wellbeing post-cancer treatment.

Interestingly, the majority of participants were unaware of the prospect of PF dysfunction following breast cancer treatment. Similar comments were made in a qualitative study exploring the experiences of genitourinary symptoms in women with breast cancer [15] and incontinence in women with gynaecological cancer [26, 29]. Participants reported that the impacts of PF dysfunction may not have been as burdensome if they were educated on the possibility of developing PF dysfunction following cancer treatment [15, 26]. This suggests it may be important to provide information regarding pelvic floor dysfunction (including potential treatment options) to women prior to starting their cancer treatments, as suggested by participants in this study. This can be done by including information about PF dysfunction in resources often used by women with breast cancer such as brochures, support group websites and cancer support gift packs. This strategy may help to alleviate the embarrassment participants reported around the disclosure of PF symptoms to health care professionals, if they know these symptoms may be related to cancer treatment [30, 31]; this lack of awareness was a barrier to the uptake of treatment for PF dysfunction amongst our participants. Even with disclosure of PF symptoms, participants felt that health care professionals preferentially prioritise breast cancer recovery rather than the management of PF symptoms. A possible reason for the lack of focus on PF symptoms by health care professionals is because the treatment of UI and FI does not currently appear in the breast cancer treatment guidelines [9, 10]. More awareness and a clear referral pathway to treatment for PF symptoms (e.g. referral to PF physiotherapists) for medical professionals to access may better address PF dysfunction in women with breast cancer.

Participants in this study expressed that PF physiotherapy (e.g. PF muscle training) to treat PF dysfunction should be integrated into existing breast cancer physiotherapy treatments. Women with breast cancer may access physiotherapy to manage other side effects of cancer treatment such as lymphoedema, cancer-related pain and fatigue, scar tissue, limited shoulder range of movement and body deconditioning after cancer treatment [32]. In this study, the majority of participants wanted to access treatment for PF dysfunction after primary cancer treatment when other side effects of cancer treatment generally develop, as they tend to access physiotherapy services at this point in their cancer recovery. Therefore, the integration of PF physiotherapy into existing breast cancer physiotherapy programmes may provide better opportunities for the disclosure and uptake of treatment for PF dysfunction in this population.

### Limitations

There are a number of limitations worth noting. Women who were bothered by their PF dysfunction may have been more likely to participate in this study. Furthermore, as interviews were conducted over videoconferencing (Zoom™), women who did not have access to internet facilities may not have been able to participate in this study and COVID-19 regulations limited our ability to offer an in-person alternative. Previous studies on the use of videoconferencing for qualitative data collection have reported some disadvantages including difficulty in building rapport with participants, having to navigate participants through technical difficulties and retaining interest for the interview being conducted [33]. In this study, we provided information about the study and Zoom™ instructions prior to the interview to mitigate technological difficulties. We also believe this strategy helped to maintain interest in participating in the interview. Future research is needed to investigate strategies to enhance rapport when using videoconferencing for qualitative data collection, especially when working with participants with breast cancer who may be embarrassed about discussing their experiences of PF dysfunction. Participants may not have been forthcoming with some information due to family members being present at home during the interview. There may also be an element of underreporting or overreporting due to recall bias when asking participants to recall their experiences from the past, especially during breast cancer treatment which can impact on the cognition and long-term memory of participants [34]. Furthermore, as the COM-B model was used to structure our findings, a truly inductive approach without this framework might have yielded different interpretations of the findings. Nevertheless, this study captured the experiences of women at a range of stages of breast cancer recovery. The consistent response that there needs to be more aware of PF dysfunction in women with breast cancer highlighted an area for clinical practice improvement in the care for this population.

## Conclusion

Participants in our study were either resigned to or bothered by their PF dysfunction. They would like to receive information about PF dysfunction prior to starting cancer treatment, be screened for PF dysfunction during cancer treatment and be offered therapies for their PF dysfunction after primary cancer treatment. Barriers to accessing treatment for PF dysfunction included a lack of awareness about PF dysfunction following breast cancer treatment and health care professionals not focussing on the management of PF symptoms during cancer treatment. Enablers included motivation to resume their normal pre-cancer lives, already accessing physiotherapy as a part of breast cancer treatment and being interested in telehealth. Further studies are needed to determine the effectiveness of treatment of PF dysfunction (e.g. PF muscle training) in order to ameliorate the effects of PF dysfunction on the lives of women with breast cancer.

## Data Availability

Not available.

## Appendix 1 Semi-structured interview guide

- Tell me about your experience with accidental loss of bladder/bowel control?
- Are you happy to discuss aspects of your current sexuality/sexual activity?
- Have you ever spoken to a health care professional about your incontinence or concerns about sexual activity?
- Did you receive any treatment for your incontinence or concerns about sexual activity?
- Do you think it is important to offer treatment for incontinence or concerns about sexual activity for women with breast cancer?
- Do you know what pelvic floor physiotherapy is?
- If no, pelvic floor physiotherapy aims to improve the function of pelvic floor muscles in order to treat incontinence and aspects of sexual dysfunction through exercises and lifestyle changes.
  - Looking back was there a particular point in time incontinence or sexual dysfunction would have been ideal to talk to a health professional about?
- When and how could information about incontinence or sexual dysfunction be better communicated to women who have been diagnosed with breast cancer?
- For women diagnosed with breast cancer at what point do you think would be best for pelvic floor physiotherapy to be offered?
  - What is your motivation (enablers) in receiving pelvic floor physiotherapy for these symptoms?
- What are some strategies that we can implement to make this treatment possible?
  - What may be some barriers to receiving pelvic floor physiotherapy for these symptoms?
- How can health care professionals help you overcome these barriers?
